# High-Frequency Intraluminal Ultrasound Versus Esophagogastroduodenoscopy for Predicting Variceal Hemorrhage After Prophylactic Endoscopic Variceal Ligation

**DOI:** 10.3390/diagnostics16172836

**Published:** 2026-09-03

**Authors:** Hana Park, Jeong Hwan Kim, Won Hyeok Choe, So Young Kwon, Jeong Han Kim, Young Koog Cheon, Tae Yoon Lee, Sang Hoon Lee, Se Min Kim, Joo Hye Song, Sun-Young Lee, In-Kyung Sung

**Affiliations:** 1 Department of Internal Medicine, Konkuk University School of Medicine, Konkuk University Hospital, Seoul 05030, Republic of Korea; hnpark80@gmail.com (H.P.); 20050101@kuh.ac.kr (W.H.C.); sykwonmd@kuh.ac.kr (S.Y.K.); jhkim@kuh.ac.kr (J.H.K.); cyk@kuh.ac.kr (Y.K.C.); 20070371@kuh.ac.kr (T.Y.L.); 20200180@kuh.ac.kr (S.H.L.); 20190173@kuh.ac.kr (S.M.K.); 20230514@kuh.ac.kr (J.H.S.); sunyoung@kuh.ac.kr (S.-Y.L.); inksung@kuh.ac.kr (I.-K.S.); 2 Digestive Disease Center, Konkuk University Hospital, Seoul 05030, Republic of Korea

**Keywords:** esophageal varices, variceal hemorrhage, endoscopic variceal ligation, endoscopic ultrasonography, primary prophylaxis

## Abstract

**Background/Objectives:** The role of endoscopic ultrasonography (EUS) in surveillance after endoscopic variceal ligation (EVL) for primary prophylaxis of esophageal variceal hemorrhage remains uncertain. This study compared the predictive value of high-frequency intraluminal ultrasound (HFIUS) and esophagogastroduodenoscopy (EGD) for subsequent variceal hemorrhage after prophylactic EVL. **Methods:** In this retrospective study, follow-up EGD and HFIUS were performed in 40 patients with liver cirrhosis who underwent EVL as primary prophylaxis against variceal hemorrhage. EGD-based variceal grade and variceal cross-sectional area (CSA) measured by HFIUS were compared in all 40 patients. Among them, 31 who achieved variceal eradication or reduction to grade 0/1 on surveillance EGD were subsequently followed to evaluate subsequent variceal hemorrhage and clinical outcomes. **Results:** Spearman’s correlation analysis revealed a statistically significant yet weak positive correlation between EGD-based variceal grade and variceal CSA measured by HFIUS (ρ = 0.347, *p* = 0.028). Among the 31 patients with grade 0/1 varices on follow-up EGD, the mean follow-up duration was 32.4 ± 8.8 months, during which variceal hemorrhage occurred in seven patients and five patients died. In this exploratory analysis based on seven hemorrhagic events, a preliminary variceal CSA cutoff of 9.8 mm^2^ predicted subsequent hemorrhage with an apparent sensitivity of 85.7% and specificity of 91.7% (optimism-corrected 75.3% and 90.0%; bootstrap-corrected AUC 0.945). Multivariate analysis showed that variceal CSA measured by HFIUS was independently associated with variceal hemorrhage (OR, 1.302; 95% CI, 1.040–2.020; *p* = 0.016), whereas EV grade assessed by surveillance EGD was not predictive of variceal hemorrhage. **Conclusions:** HFIUS-derived variceal CSA was associated with subsequent hemorrhage among patients with grade 0/1 varices after EVL and may provide prognostic information not captured by endoscopic grade alone. Incorporation of HFIUS-based EUS into post-EVL surveillance may help identify patients at persistent bleeding risk who may benefit from intensified prophylactic strategies, even when grade 0/1 eradication has been confirmed on surveillance EGD. Given the small number of events, the proposed cutoff is preliminary and requires external validation.

## 1. Introduction

Esophageal variceal hemorrhage develops at an annual incidence of approximately 5–15% among patients with liver cirrhosis and esophageal varices [1,2]. Established risk factors for bleeding include large varices, advanced hepatic decompensation, and red color (RC) signs identified on endoscopy [3,4]. Accordingly, primary prophylactic treatment is recommended for patients considered to be at high risk for variceal bleeding [5,6].

Nonselective beta-blockers and endoscopic variceal ligation (EVL) are widely accepted approaches for primary prophylaxis of esophageal variceal hemorrhage [7,8,9,10], and combination therapy may be used in selected patients. Although previous studies have generally shown that combined EVL and nonselective beta-blocker therapy does not provide significant advantages in bleeding prevention or survival compared with monotherapy and is therefore not routinely recommended [11,12], several reports have suggested that combination therapy may reduce the risk of initial bleeding episodes and post-eradication recurrence [13]. Nevertheless, clear criteria for identifying patients who may benefit from combination treatment have not yet been established.

EVL is commonly repeated until variceal eradication is achieved, usually defined as complete disappearance of varices or reduction to varices too small for additional ligation, corresponding to EV grade 0 or 1 [14]. Post-treatment surveillance is conventionally performed using esophagogastroduodenoscopy (EGD) [7,8,9], which evaluates luminal appearance but may not fully reflect residual submucosal variceal burden. Previous observational studies have suggested that endoscopic ultrasonography (EUS) can detect residual varices and persistent collateral blood flow not visible on conventional endoscopy [15,16,17], raising the possibility that EUS-based assessment may provide prognostically relevant information beyond standard endoscopic grading. However, whether EUS findings after prophylactic EVL can predict subsequent variceal hemorrhage remains to be established.

Therefore, in the present study, we first assessed the correlation between EGD-based variceal grades and variceal cross-sectional area (CSA) measured by high-frequency intraluminal ultrasound (HFIUS) in patients who underwent EVL as primary prophylaxis. We then evaluated, in an exploratory analysis, whether HFIUS-derived variceal CSA could predict subsequent variceal hemorrhage in patients who achieved variceal eradication or reduction to grade 0/1 on surveillance EGD, and compared its predictive value with that of conventional EGD-based grading.

## 2. Materials and Methods

### 2.1. Study Protocol

This retrospective, single-center study was conducted at Konkuk University Hospital, Seoul, Korea. Consecutive patients who underwent endoscopic variceal ligation between January 2019 and January 2021 were eligible; this interval refers to the period during which the index procedures were performed, and outcomes were subsequently ascertained from the medical records. The Institutional Review Board of Konkuk University Hospital determined that the protocol met the institutional criteria for exemption from review and granted a waiver of informed consent owing to the retrospective design (protocol code KUMC 2026-07-029; date of determination 15 July 2026). Under the terms of that determination, the study could commence only after the determination date; the retrospective review of medical records was accordingly undertaken in July 2026, and no patient records were accessed or extracted beforehand. A total of 40 consecutive patients with liver cirrhosis who underwent one to four sessions of EVL as primary prophylaxis against variceal hemorrhage were retrospectively identified. Clinical, endoscopic, and follow-up data were collected from the patients’ medical records. Patients with advanced hepatocellular carcinoma (HCC) or decompensated liver cirrhosis (Child–Pugh class C) were excluded. In accordance with our institutional surveillance policy, follow-up EGD was scheduled approximately three months after completion of the final EVL session, and HFIUS was performed synchronously at that examination (actual interval, 1–4 months). Among the 40 patients, 31 achieved variceal eradication or reduction to grade 0/1 according to EGD findings and were subsequently followed to evaluate variceal hemorrhage and clinical outcomes. Because variceal eradication or reduction to grade 0/1 had been achieved, nonselective beta-blockers were not administered unless variceal hemorrhage developed during follow-up. Variceal hemorrhage was defined as hematemesis, hematochezia, or melena with endoscopically confirmed bleeding from esophageal varices. The primary aim of this study was to evaluate the correlation between EGD-based EV grades and HFIUS measurements in the 40 patients who underwent both examinations. The secondary aim was to assess the predictive value of EGD grades and HFIUS measurements for subsequent variceal hemorrhage in the 31 patients who underwent follow-up after variceal eradication or reduction to grade 0/1. This analysis was deliberately restricted to patients who had achieved grade 0/1 status, because the clinical question it addresses—whether surveillance alone is sufficient once eradication appears complete—arises only at that point. Patients who did not achieve eradication remained under active variceal therapy and were not followed for this endpoint.

### 2.2. Diagnostic and Therapeutic Endoscopy

EVL was performed using either a pneumatic-active ligating device (Samjin, Seoul, Republic of Korea) or a multiband ligator (Saeed Six Shooter; Cook Endoscopy, Winston-Salem, NC, USA). The bleeding site and surrounding varices were ligated as extensively as possible. Follow-up EGD was performed using a double-channel endoscope (GIF2T-240; Olympus, Tokyo, Japan), and images were electronically recorded for review by two endoscopists. Post-EVL varices were graded according to the Japanese Research Society for Portal Hypertension as follows [18,19]: grade 0, not visible; grade 1, small and straight; grade 2, enlarged and tortuous; and grade 3, large or coil-shaped. RC signs were classified as positive or negative. Positive RC signs included cherry-red spots, red wale markings, or hemocystic spots.

### 2.3. High-Frequency Intraluminal Ultrasound Examination

**Figure 1:**
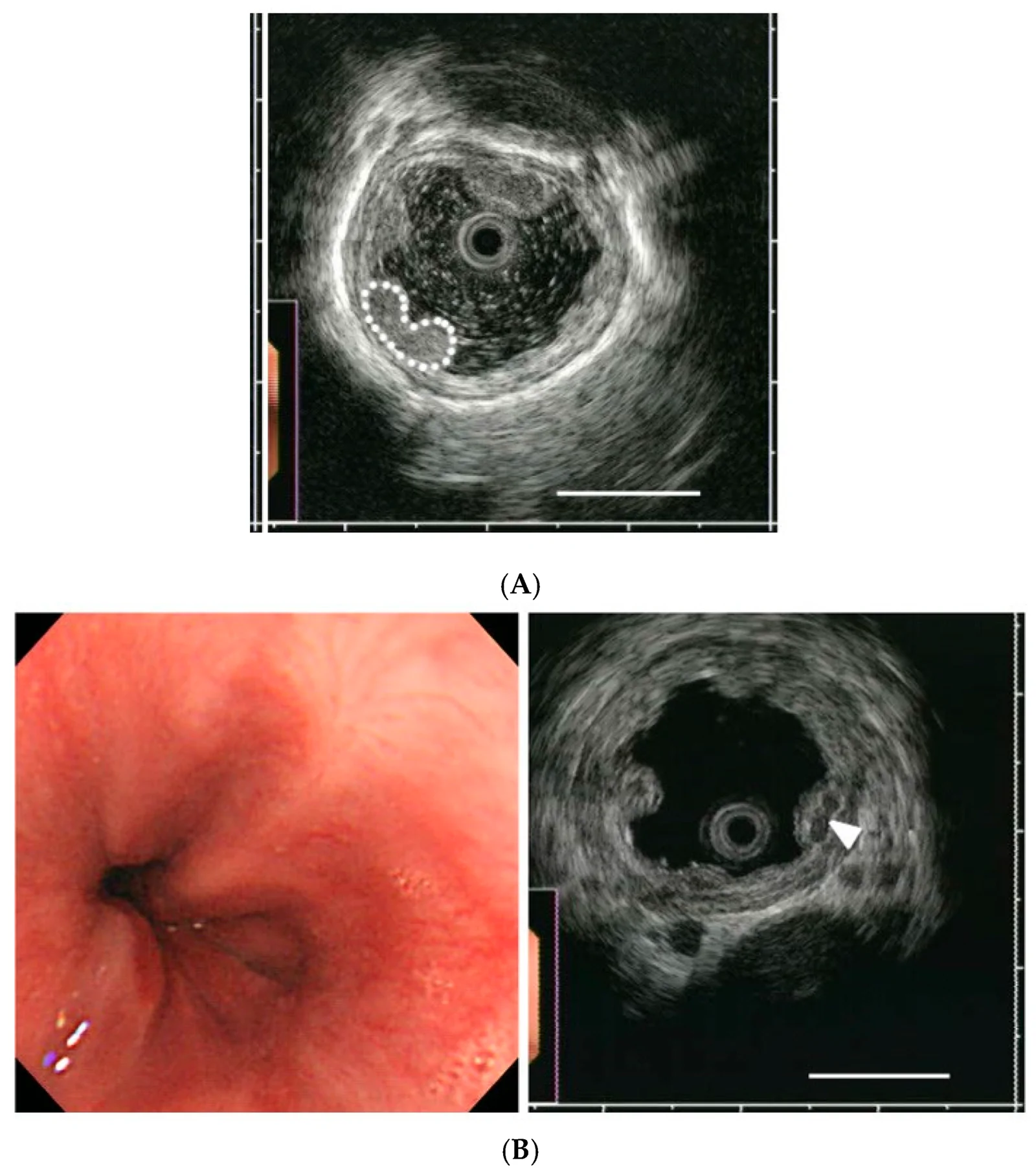
(**A**) Endoscopic ultrasonography (EUS) image using high-frequency intraluminal ultrasound (HFIUS) of post-endoscopic variceal ligation (EVL) esophageal varices. The dotted line indicates the largest variceal cross-sectional area (CSA). Scale bar = 10 mm. (**B**) The varix was classified as grade 0 on esophagogastroduodenoscopy (EGD), whereas EUS using HFIUS demonstrated a residual variceal CSA of 6.23 mm^2^. The arrow indicates the residual varix detected by HFIUS.

HFIUS was performed simultaneously with EGD using a 2.3-millimeter ultrasonic miniprobe with a 20-megahertz transducer (UM-G20-29R; Olympus). The probe was inserted through an accessory channel, while deaerated water was infused through the other channel. Air insufflation was minimized to avoid compression of the varices. The probe was advanced to the stomach and gradually withdrawn to scan the distal third of the esophagus. All varices visualized during withdrawal were assessed, and the single varix with the largest apparent CSA was selected for measurement. CSA was measured in real time during the examination using ImageJ software (version 1.54f; NIH, Bethesda, MD, USA) by two experienced examiners (JHK and WHC) in consensus [20], and images were electronically recorded (Figure 1A). CSA was defined as the area between the hypoechoic blood-filled lumen and the hyperechoic mucosal or submucosal layer (Figure 1B). Because measurements were obtained contemporaneously with the procedure, examiners were necessarily unaware of subsequent hemorrhage outcomes; they were, however, aware of the concurrent EGD findings, as HFIUS was performed during the same session. Individual measurements from each examiner were not archived.

### 2.4. Statistical Analysis

Continuous variables were expressed as mean ± standard deviation and were compared using Student’s *t*-test or the Mann–Whitney U test. Categorical variables were analyzed using the χ^2^ test or Fisher’s exact test, as appropriate. Correlations between EGD grades and HFIUS measurements were evaluated using Spearman’s correlation coefficient. Because several candidate predictors exhibited complete or quasi-complete separation (for example, no variceal hemorrhage occurred among patients with current or previously treated HCC), univariate and multivariate associations with subsequent variceal hemorrhage and mortality were assessed using Firth’s penalized-likelihood logistic regression, with odds ratios (OR), 95% confidence intervals (CI), and *p* values derived from the profile penalized likelihood. Variables that were statistically significant in univariate analysis were entered into the multivariate model. Receiver operating characteristic (ROC) curve analysis was performed to identify predictors of variceal hemorrhage and determine optimal cutoff values. Internal validation was performed by bootstrap optimism correction with 2000 resamples, in which the entire modeling procedure, including selection of the Youden-optimal threshold, was repeated within each resample; threshold stability was summarized by the distribution of Youden-optimal cutoffs across resamples. Leave-one-out cross-validation was performed as a secondary internal validation.

Logistic regression was used as the primary analysis because the question concerned the probability of hemorrhage within the observation window rather than the instantaneous hazard over time. Because death without preceding hemorrhage precludes observation of the event of interest, cumulative incidence was estimated using the Aalen–Johansen estimator and subdistribution hazard ratios using the Fine–Gray model, with cause-specific Cox regression and a permutation test (2000 permutations) as sensitivity analyses.

Statistical analyses were performed using IBM SPSS Statistics version 27.0 (IBM Corp., Armonk, NY, USA); Firth’s penalized-likelihood logistic regression was performed using the logistf package (version 1.26.1) in R version 4.4.1 (R Foundation for Statistical Computing, Vienna, Austria). Internal validation, competing-risk analyses, and the sensitivity analyses were performed in Python 3.12.3 (lifelines 0.30.3), with Firth’s penalized likelihood and the Fine–Gray model implemented directly and verified against the primary estimates. A two-tailed *p* value < 0.05 was considered statistically significant.

The prognostic analyses were based on 31 patients with seven hemorrhagic events, corresponding to 3.5 events per variable in the multivariable model; all prognostic estimates were therefore regarded as exploratory.

## 3. Results

### 3.1. Patient Characteristics and Clinical Outcomes

A total of 40 patients with liver cirrhosis who had undergone EVL as primary prophylaxis for esophageal variceal hemorrhage were included in this study. Follow-up assessments were performed using EGD and HFIUS-based EUS. Patients underwent a mean of 2.0 ± 0.7 EVL sessions, with a mean of 3.3 ± 0.5 band ligations performed per session. Baseline characteristics are summarized in Table 1.

Following EVL, variceal eradication or reduction to grade 0/1 was achieved in 31 patients—complete eradication to grade 0 in 23 and reduction to grade 1 in 8—whereas the remaining 9 patients did not achieve this endpoint. The two groups were comparable in age, Child–Pugh score, MELD score, and laboratory parameters, but variceal CSA measured by HFIUS was significantly larger in patients who did not achieve eradication (14.3 ± 7.9 versus 6.7 ± 6.7 mm^2^, *p* = 0.010; Table 1). Male sex was also more frequent among those achieving eradication (*p* = 0.034), although this comparison rests on only three female patients in that group. Subsequent analyses of variceal hemorrhage were confined to the 31 patients who achieved eradication or reduction to grade 0/1; the mean follow-up duration in these patients was 32.4 ± 8.8 months (median 30, range 13–48).

During follow-up, variceal hemorrhage occurred in 7 patients, with a mean interval to bleeding of 17.0 ± 6.2 months. A total of five patients died during follow-up, including one death related to variceal hemorrhage, two related to HCC, and two related to decompensated cirrhosis.

### 3.2. Correlation Between EUS-Based Variceal CSA and EGD Variceal Grades

**Figure 2:**
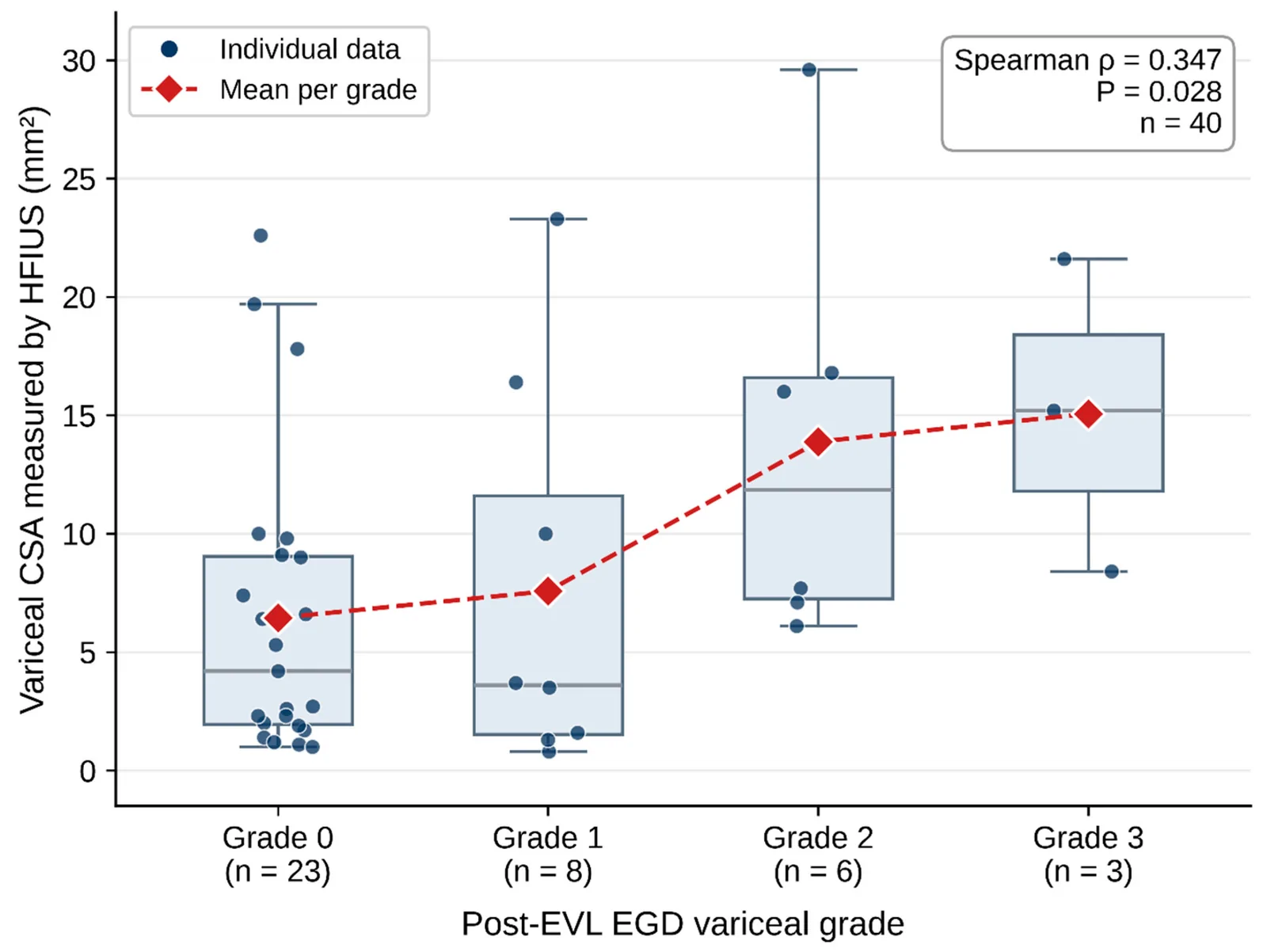
Correlation between esophagogastroduodenoscopy (EGD)-based variceal grade and variceal cross-sectional area (CSA) measured by high-frequency intraluminal ultrasound (HFIUS). Each blue dot represents an individual patient (*n* = 40), with horizontal jitter applied to reduce over-plotting. Boxes indicate the median and interquartile range, and the red diamonds connected by the dashed line indicate the mean CSA for each variceal grade. A statistically significant yet weak positive correlation was observed between EGD-based variceal grade and variceal CSA (Spearman’s ρ = 0.347, *p* = 0.028).

Among the 40 patients who underwent EVL as primary prophylaxis, follow-up EGD demonstrated variceal grades 0, 1, 2, and 3 in 23, 8, 6, and 3 patients, respectively. The mean largest variceal CSA measured by HFIUS was 6.4 ± 6.2 mm^2^ in patients with grade 0 varices, 7.6 ± 8.3 mm^2^ in those with grade 1 varices, 13.9 ± 9.0 mm^2^ in those with grade 2 varices, and 15.1 ± 6.6 mm^2^ in those with grade 3 varices (Figure 2).

Spearman’s correlation analysis demonstrated a statistically significant but only weak positive correlation between EGD-based variceal grade and variceal CSA measured by HFIUS (ρ = 0.347, *p* = 0.028), indicating only a weak association between the two measures. Nevertheless, the mean variceal CSA was significantly larger in patients with EGD grades 2/3 varices than in those with grades 0/1 varices (14.3 ± 7.9 mm^2^ vs. 6.7 ± 6.7 mm^2^, *p* = 0.010).

### 3.3. Predictive Value of EUS-Based Variceal CSA for Variceal Hemorrhage

**Figure 3:**
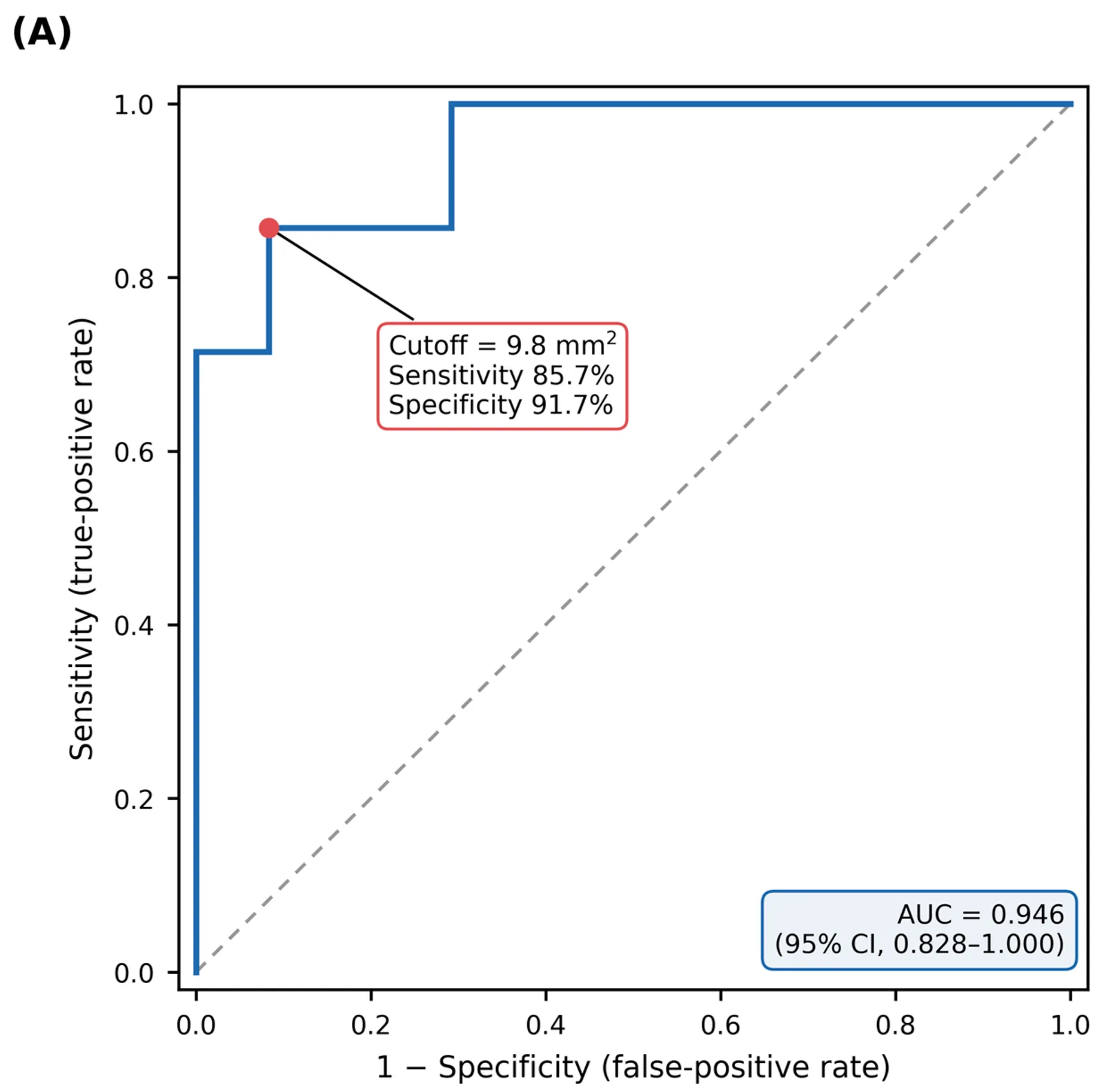

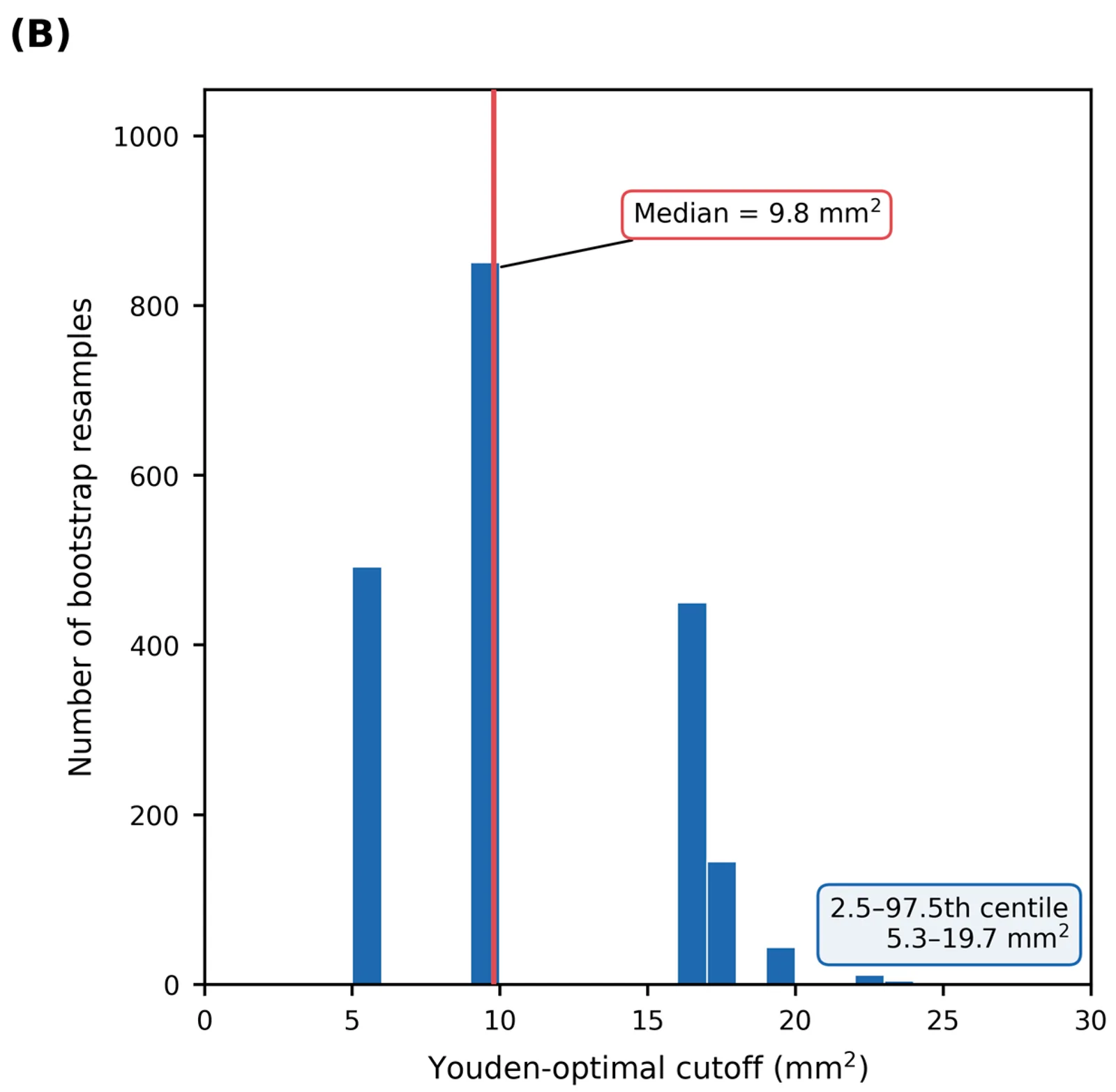
Receiver operating characteristic (ROC) curve of the maximum variceal cross-sectional area (CSA) measured by high-frequency intraluminal ultrasound (HFIUS) for predicting subsequent variceal hemorrhage among the 31 patients who achieved variceal eradication or reduction to grade 0/1 after endoscopic variceal ligation (EVL). (**A**) ROC curve. The area under the curve (AUC) was 0.946 (95% CI, 0.828–1.000). A CSA cutoff of 9.8 mm^2^ (red point) yielded a sensitivity of 85.7% and a specificity of 91.7%. The diagonal dashed line represents the reference line of no discrimination (AUC = 0.5). (**B**) Distribution of the Youden-optimal cutoff across 2000 bootstrap resamples. The median was 9.8 mm^2^ (red line), with a 2.5th–97.5th centile range of 5.3 to 19.7 mm^2^.

Among the 31 patients who achieved variceal eradication or reduction to grade 0/1 on follow-up EGD, the largest variceal CSA was significantly greater in the 7 patients who subsequently developed variceal hemorrhage than in the 24 patients who did not (16.41 ± 6.66 mm^2^ vs. 3.90 ± 3.14 mm^2^, respectively; *p* < 0.001). On ROC analysis based on seven hemorrhagic events, the largest variceal CSA showed a high apparent area under the curve (AUC, 0.946; 95% CI, 0.828–1.000; Figure 3A), and the Youden-optimal cutoff of 9.8 mm^2^ yielded an apparent sensitivity of 85.7% and specificity of 91.7%. Because this threshold was both derived and evaluated in the same cohort, it is presented as a preliminary value.

On internal validation by bootstrap resampling, discrimination was minimally optimistic: the optimism-corrected AUC was 0.945 (estimated optimism 0.001), and leave-one-out cross-validation gave an AUC of 0.911. The threshold-dependent measures were more optimistic, with corrected sensitivity 75.3%, specificity 90.0%, and accuracy 86.8% (Supplementary Table S1a). The Youden-optimal cutoff had a median of 9.8 mm^2^ across resamples and was selected exactly in 42.6% of them, but the 2.5th-to-97.5th centile range extended from 5.3 to 19.7 mm^2^ (Figure 3B), indicating that the threshold is recovered as a central estimate while remaining imprecise.

**Figure 4:**
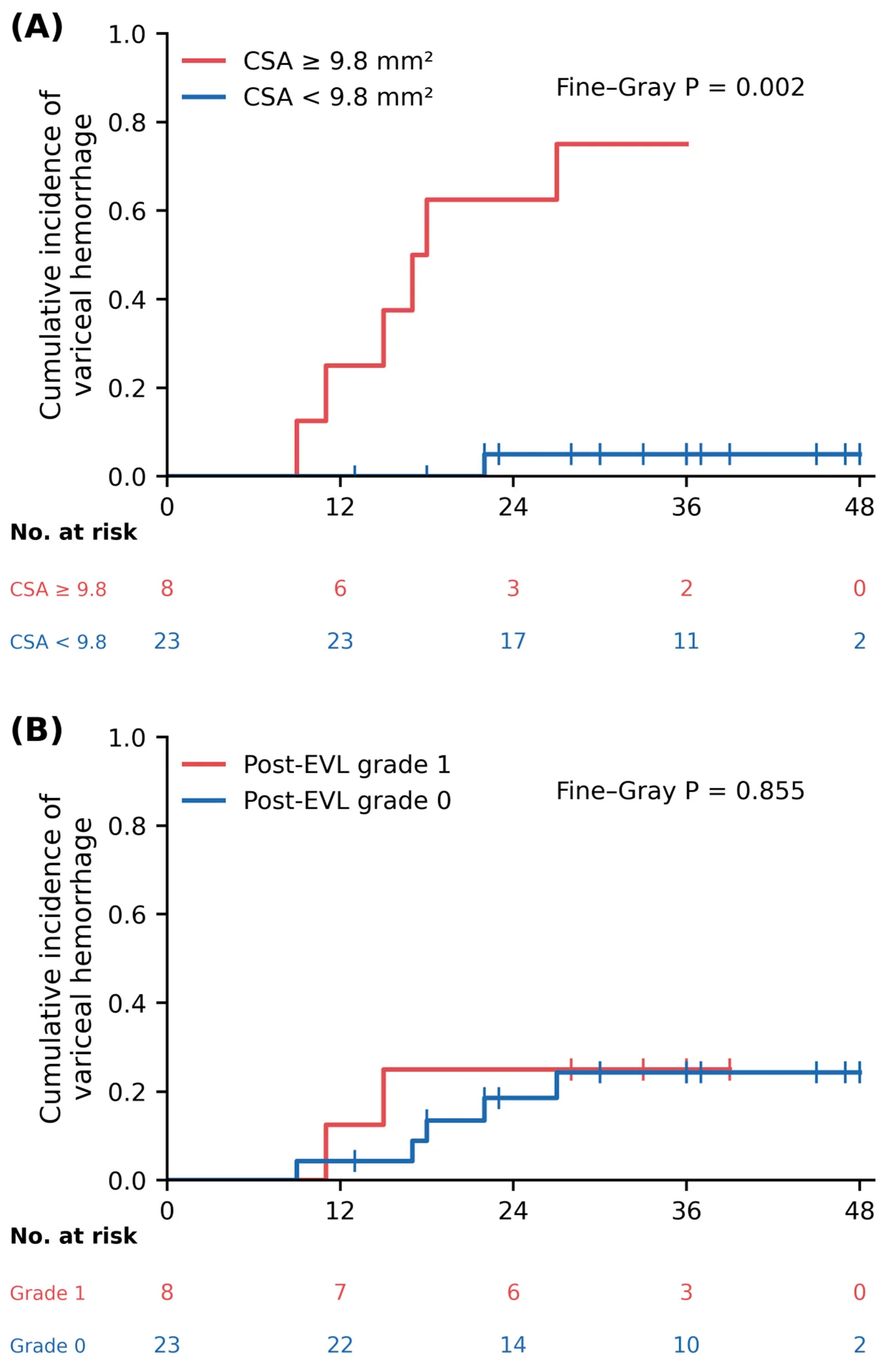
Cumulative incidence of variceal hemorrhage, estimated with the Aalen–Johansen estimator with death without preceding hemorrhage treated as a competing event. (**A**) Stratified by variceal cross-sectional area (CSA) measured by high-frequency intraluminal ultrasound: CSA ≥ 9.8 mm^2^ (*n* = 8) versus < 9.8 mm^2^ (*n* = 23); Fine–Gray *p* = 0.002. (**B**) Stratified by post-EVL variceal grade on EGD: grade 1 (*n* = 8) versus grade 0 (*n* = 23); Fine–Gray *p* = 0.855. Tick marks indicate censored observations.

Treating death without preceding hemorrhage (*n* = 4) as a competing event, the cumulative incidence of variceal hemorrhage at 36 months was 75.0% in patients with a CSA ≥ 9.8 mm^2^ versus 5.0% in those below the threshold (Fine–Gray subdistribution hazard ratio 27.16, 95% CI 3.24–228.0, *p* = 0.002; Figure 4A), whereas no difference was observed by post-EVL variceal grade (25.0% versus 24.4%; *p* = 0.855; Figure 4B). Analyzed continuously, CSA remained associated with hemorrhage (subdistribution hazard ratio 1.337 per mm^2^, 95% CI 1.123–1.593, *p* = 0.001). Cause-specific Cox regression yielded numerically identical estimates, as all competing deaths occurred later than the last observed hemorrhage.

### 3.4. Predictors of Subsequent Variceal Hemorrhage and Overall Mortality

Among the baseline clinical and endoscopic characteristics of the 31 patients—including age, sex, etiology of cirrhosis, Child–Pugh score, current or previously treated HCC, variceal grade on initial EGD, presence of RC signs on initial EGD, maximum variceal CSA by HFIUS-EUS, variceal grade on follow-up EGD after EVL, and RC signs on follow-up EGD after EVL—univariate analysis identified the presence of RC signs on initial EGD (OR, 26.619; 95% CI, 3.979–324.892; *p* < 0.001) and the maximum variceal CSA by HFIUS-EUS (OR, 1.407; 95% CI, 1.160–2.145; *p* < 0.001) as significant predictors of subsequent variceal hemorrhage. When patients were dichotomized at the 9.8 mm^2^ cutoff, those with a CSA ≥ 9.8 mm^2^ had a markedly higher risk of hemorrhage than those with a CSA <9.8 mm^2^ (OR, 39.0; 95% CI, 5.375–526.047; *p* < 0.001). With only seven events, the confidence intervals for the dichotomized CSA and for RC signs on initial EGD span more than two orders of magnitude; these point estimates should be interpreted as indicating the direction and consistency of the association rather than its magnitude. In contrast, variceal hemorrhage was not significantly associated with variceal grade on follow-up EGD after EVL (OR, 1.294; 95% CI, 0.194–7.115; *p* = 0.774) or with RC signs on follow-up EGD after EVL (OR, 3.615; 95% CI, 0.261–50.704; *p* = 0.309). In the multivariate analysis, the maximum variceal CSA by HFIUS-EUS remained the only independent predictor of subsequent variceal hemorrhage (OR, 1.302; 95% CI, 1.040–2.020; *p* = 0.016) (Table 2). To quantify the incremental value of CSA over the strongest conventional endoscopic predictor, the two models were compared directly: adding CSA to a model containing initial RC signs improved model fit (penalized likelihood ratio test χ^2^ = 5.77, df = 1, *p* = 0.016) and raised the c-statistic from 0.866 to 0.952, whereas adding RC signs to a model containing CSA did not (χ^2^ = 0.71, df = 1, *p* = 0.401). The lowest AIC (11.96) and the highest leave-one-out cross-validated AUC (0.911) were both obtained with CSA alone (Supplementary Table S1b), consistent with overfitting of the two-predictor model at 3.5 events per variable.

Among the baseline characteristics of the 31 patients, Child–Pugh score was the only significant predictor of mortality on univariate analysis (OR, 2.671; 95% CI, 1.300–7.683; *p* = 0.006). Current or previously treated HCC was not significantly associated with mortality (OR, 4.796; 95% CI, 0.616–36.730; *p* = 0.128). In the multivariate analysis, Child–Pugh score remained the only independent predictor (OR, 2.501; 95% CI, 1.236–6.812; *p* = 0.009) (Table 3).

## 4. Discussion

In the present study, EUS-based measurement of variceal CSA using HFIUS was significantly associated with subsequent variceal hemorrhage after primary prophylactic EVL, whereas EV grades determined by conventional EGD were not predictive of later bleeding events after apparent variceal eradication. Furthermore, only a weak correlation was observed between EGD-based EV grades and HFIUS-derived variceal CSA, suggesting that HFIUS may provide additional information regarding residual variceal burden beyond that obtained from conventional endoscopic grading alone.

EVL is widely accepted as an effective treatment for primary prophylaxis of esophageal variceal hemorrhage, and reduction of varices to grade 0/1 on EGD is generally regarded as a satisfactory therapeutic endpoint [14,21]. Nevertheless, several patients in the present study experienced subsequent variceal hemorrhage despite apparent eradication on surveillance EGD. This observation suggests that residual submucosal varices or persistent collateral circulation may remain even when varices appear eradicated on conventional endoscopy [22]. Previous studies have also demonstrated that endoscopic ultrasonography can detect residual varices and collateral vessels after EVL that may not be visible during standard endoscopic examination [15,16,17,23,24]. Our results further support the potential role of quantitative HFIUS assessment in identifying persistent hemodynamic risk following apparent endoscopic eradication.

Current guidelines recommend either nonselective beta-blockers or EVL for primary prophylaxis in patients with high-risk esophageal varices [7,8,9,10]. Although routine combination therapy is generally not recommended [11,12], some reports have suggested that additional pharmacologic therapy may be beneficial in selected high-risk patients [7,13]. However, reliable methods for identifying these patients remain limited. In the present study, patients with larger residual variceal CSA measured by HFIUS showed a significantly increased risk of subsequent variceal hemorrhage despite apparent eradication to grade 0/1 on surveillance EGD. These findings suggest that HFIUS-based assessment may help identify patients who remain at elevated bleeding risk after EVL. Whether such patients would benefit from more intensive preventive treatment, including nonselective beta-blockers, was not evaluated in this study; because beta-blockers were not administered after eradication in this cohort, our data established a prognostic association only, and this question would require a prospective interventional trial. Although EGD remains the standard surveillance method after variceal ligation [7,8,9], endoscopic grading evaluates luminal appearance and may not fully reflect persistent submucosal variceal burden. In contrast, HFIUS allows direct visualization and quantitative assessment of residual variceal structures, potentially providing clinically relevant prognostic information beyond conventional endoscopy during post-EVL surveillance.

The threshold identified in this cohort requires careful interpretation. Internal validation indicated that discrimination was not materially optimistic, with an optimism-corrected AUC of 0.945 and a leave-one-out estimate of 0.911. The threshold itself, however, was imprecise: although a cutoff of 9.8 mm^2^ was recovered as the central estimate across bootstrap resamples, the 2.5th-to-97.5th centile range extended from 5.3 to 19.7 mm^2^, and the sensitivity attached to it fell from 85.7% to 75.3% after optimism correction. The 9.8 mm^2^ value should therefore be regarded as a preliminary cutoff derived in this cohort rather than a validated decision boundary, and external validation in an independent population is required before it could inform management. Although the multivariable model indicated that CSA was associated with hemorrhage independently of RC signs, adding RC signs to a model containing CSA did not improve fit, and cross-validation favored the single-predictor model (Supplementary Table S1b); CSA alone is therefore likely to provide the more robust basis for any future prediction rule.

This study has several limitations. First, it was a single-center, retrospective observational study of 31 patients with only seven hemorrhagic events, and all prognostic estimates should be regarded as exploratory. The multivariable model was fitted with 3.5 events per variable, well below conventional recommendations, and the resulting confidence intervals are correspondingly wide. In this cohort all competing deaths occurred after the last observed hemorrhage, so competing-risk and Kaplan–Meier estimates coincided; this concordance should not be expected with longer follow-up or higher liver-related mortality. Second, the analysis was restricted by design to patients who had achieved grade 0/1 status, so the proposed threshold should not be applied to patients with persistent varices. Child–Pugh class C patients were not represented, and residual variceal burden may carry greater prognostic weight in decompensated cirrhosis. Hemorrhage outcomes were unavailable for the nine patients who did not achieve eradication, although their cross-sectional areas were substantially larger, consistent with the measurement reflecting true residual burden; imputing their outcomes across all possible scenarios showed that the association persisted even under the maximally adverse assignment (Supplementary Table S2), although these outcomes are hypothetical. Third, HFIUS measurements were performed by experienced examiners at a tertiary referral center, and because individual readings were not archived, interobserver agreement could not be quantified; a simulation assuming intraclass correlation coefficients as low as 0.70 indicated that the association would persist (Supplementary Table S3). Examiners were aware of the concurrent endoscopic grade, although the weak correlation observed between grade and CSA (Spearman’s ρ = 0.347; Figure 2) argues against systematic adjustment of measurements toward the endoscopic impression. Measured agreement and reproducibility in general practice remain to be established. Fourth, nonselective beta-blockers were not administered after variceal eradication unless hemorrhage developed. The study therefore establishes a prognostic association only and cannot determine whether identifying patients with a large residual CSA and treating them differently would reduce subsequent hemorrhage. Fifth, although follow-up endoscopy was scheduled at approximately three months in accordance with our institutional surveillance policy, the actual interval ranged from one to four months, reflecting real-world variations in outpatient visit scheduling rather than strict prospective timing. Because variceal remodeling and regression are time-dependent, this variation may have introduced confounding; however, given the limited number of events (n=7), incorporating follow-up interval as an additional covariate in the multivariable model would have resulted in severe model overfitting. Future prospective studies with standardized, fixed surveillance intervals are warranted to eliminate this potential bias. Finally, direct hemodynamic measurements such as hepatic venous pressure gradient were not available in this study.

## 5. Conclusions

HFIUS-derived variceal CSA was associated with subsequent hemorrhage among patients with grade 0/1 varices after EVL and may provide prognostic information not captured by endoscopic grade alone. Incorporation of HFIUS-based EUS into post-EVL surveillance may help identify patients at persistent bleeding risk who may benefit from intensified prophylactic strategies, including nonselective beta-blockers, even when grade 0/1 eradication has been confirmed on surveillance EGD. Because these findings are based on a small number of events, the proposed threshold should be regarded as preliminary, and large-scale prospective studies are warranted to validate it and to define optimal strategies for selecting patients for additional prophylactic treatment after EVL.

## Figures and Tables

**Table 1:** Baseline characteristics of patients who underwent EVL as primary prophylaxis, overall and according to whether variceal eradication or reduction to grade 0/1 was achieved on follow-up EGD.

Characteristics	Total (*n* = 40)	Eradication Status on Follow-Up EGD		
		Achieved (*n* = 31)	Not Achieved (*n* = 9)	*p* Value
Age, years	55.4 ± 8.6	55.5 ± 7.7	54.8 ± 11.7	0.685
Gender, male/female	33/7	28/3	5/4	0.034
Etiology of cirrhosis, alcoholic/nonalcoholic	16/24	15/16	1/8	0.061
Child–Pugh score, 5/6/7/8/9	13/10/8/6/3	12/7/4/5/3	1/3/4/1/0	0.462
MELD score	9.8 ± 2.9	9.5 ± 3.1	10.8 ± 1.8	0.160
Current or previously treated HCC, *n*	8	5	3	0.348
Platelet count, 10^3^/µL	104.0 ± 33.7	102.1 ± 33.1	110.2 ± 36.9	0.697
Serum albumin, g/dL	3.7 ± 0.6	3.8 ± 0.6	3.4 ± 0.4	0.066
Total bilirubin, mg/dL	1.6 ± 1.0	1.5 ± 1.0	1.9 ± 1.0	0.157
Prothrombin time, INR	1.10 ± 0.16	1.11 ± 0.18	1.08 ± 0.08	0.697
Variceal grade on initial EGD, grade 1/2/3	3/21/16	3/17/11	0/4/5	0.223
Presence of RC signs on initial EGD, *n*	12	9	3	1.000
No. of EVL sessions	2.0 ± 0.7	1.9 ± 0.7	2.4 ± 0.7	0.052
Variceal CSA on HFIUS, mm^2^	8.4 ± 7.6	6.7 ± 6.7	14.3 ± 7.9	0.010

Values are presented as mean ± standard deviation or number of patients (*n*). *p* values compare patients who did and did not achieve variceal eradication or reduction to grade 0/1, using the Mann–Whitney U test for continuous variables and Fisher’s exact test for categorical variables. Abbreviations: CSA, cross-sectional area; EGD, esophagogastroduodenoscopy; EVL, endoscopic variceal ligation; HCC, hepatocellular carcinoma; HFIUS, high-frequency intraluminal ultrasound; INR, international normalized ratio; MELD, model for end-stage liver disease; RC signs, red color sign.

**Table 2:** Univariate and multivariate logistic regression analyses of factors associated with subsequent variceal hemorrhage.

Variable	Non-Hemorrhage (*n* = 24)	Hemorrhage (*n* = 7)	Univariate		Multivariate	
			OR (95% CI)	*p* Value	OR (95% CI)	*p* Value
Age, years	55.6 ± 7.4	55.4 ± 9.4	0.995 (0.897–1.111)	0.931		
Gender, male/female	22/2	6/1	0.481 (0.053–5.942)	0.527		
Etiology of cirrhosis, alcoholic/nonalcoholic	12/12	3/4	0.778 (0.146–3.888)	0.758		
Child–Pugh score, 5/6/7/8/9	10/5/2/5/2	2/2/2/0/1	1.063 (0.582–1.871)	0.832		
Current or previously treated HCC, *n*	5	0	0.236 (0.002–2.542)	0.272		
Variceal grade on initial EGD, grade 1/2/3	2/13/9	1/4/2	0.703 (0.186–2.562)	0.585		
Presence of RC sign on initial EGD, *n*	3	6	26.619 (3.979–324.892)	<0.001	3.406 (0.172–63.580)	0.360
Maximum variceal CSA by HFIUS-EUS, mm^2^	3.9 ± 3.1	16.4 ± 6.7	1.407 (1.160–2.145)	<0.001	1.302 (1.040–2.020)	0.016
Variceal grade on follow-up EGD after EVL, 0/1	18/6	5/2	1.294 (0.194–7.115)	0.774		
RC sign on follow-up EGD after EVL, *n*	1	1	3.615 (0.261–50.704)	0.309		

Values are odds ratios (ORs) with 95% confidence intervals (CIs). Estimates were obtained by Firth’s penalized-likelihood logistic regression; 95% CIs and *p* values are based on profile penalized likelihood. This method was used because several predictors showed complete or quasi-complete separation (e.g., no hemorrhage occurred among patients with current or previously treated HCC), for which standard maximum-likelihood logistic regression does not yield stable estimates. Multivariate analysis included the two variables significant on univariate analysis (RC sign on initial EGD and maximum variceal CSA). Abbreviations: CI, confidence interval; CSA, cross-sectional area; EGD, esophagogastroduodenoscopy; EVL, endoscopic variceal ligation; HCC, hepatocellular carcinoma; HFIUS, high-frequency intraluminal ultrasonography; OR, odds ratio; RC sign, red color sign.

**Table 3:** Univariate and multivariate logistic regression analyses of factors associated with overall mortality.

Variable	Survivors (*n* = 26)	Non-Survivors (*n* = 5)	Univariate		Multivariate	
			OR (95% CI)	*p* Value	OR (95% CI)	*p* Value
Age, years	54.5 ± 7.5	60.8 ± 7.4	1.123 (0.979–1.366)	0.105		
Gender, male/female	24/2	4/1	0.306 (0.032–3.912)	0.325		
Etiology of cirrhosis, alcoholic/nonalcoholic	13/13	2/3	0.714 (0.104–4.300)	0.711		
Child–Pugh score, 5/6/7/8/9	11/7/4/4/0	1/0/0/1/3	2.671 (1.300–7.683)	0.006	2.501 (1.236–6.812)	0.009
Current or previously treated HCC, *n*	3	2	4.796 (0.616–36.730)	0.128	5.312 (0.422–102.591)	0.186

Values are odds ratios (OR) with 95% confidence intervals (CI). Estimates were obtained by Firth’s penalized-likelihood logistic regression, consistent with the analysis of variceal hemorrhage (Table 2); 95% CIs and *p* values are based on the profile penalized likelihood. Multivariate analysis included Child–Pugh score and current or previously treated HCC. Abbreviations: CI, confidence interval; HCC, hepatocellular carcinoma; OR, odds ratio.

## Data Availability

The raw data supporting the conclusions of this article will be made available by the authors upon reasonable request.

## Supplementary Materials

The following supporting information can be downloaded at https://www.mdpi.com/article/10.3390/diagnostics16172836/s1, Table S1a: Apparent and optimism-corrected performance of HFIUS-derived variceal cross-sectional area; Table S1b: Comparison of prediction models; Table S2: Tipping-point analysis of imputed hemorrhage outcomes; Table S3: Measurement-error sensitivity analysis.

## Abbreviations

The following abbreviations are used in this manuscript:

AUC	area under the curve
CI	confidence interval
CSA	cross-sectional area
EGD	esophagogastroduodenoscopy
EUS	endoscopic ultrasonography
EVL	endoscopic variceal ligation
HCC	hepatocellular carcinoma
HFIUS	high-frequency intraluminal ultrasound
OR	odds ratio
RC	red color (as in RC sign)
ROC	receiver operating characteristic
