# Supplementary material for: High-Frequency Intraluminal Ultrasound Versus Esophagogastroduodenoscopy for Predicting Variceal Hemorrhage After Prophylactic Endoscopic Variceal Ligation
Source: Diagnostics (Basel). 2026 Sep 3;16(17):2836. doi: 10.3390/diagnostics16172836 (PMC13564985; doi:10.3390/diagnostics16172836)
Supplement: Supplementary file 1 [file diagnostics-16-02836-s001.zip › diagnostics-4511688-supplementary.pdf]

Supplementary Materials

Table S1(a). Apparent and optimism-corrected performance of high-frequency intraluminal ultrasound-derived variceal cross-sectional area for predicting subsequent variceal hemorrhage.

Performance measure	Apparent	Estimated optimism	Optimism-corrected
Area under the ROC curve	0.946	0.001	0.945
Sensitivity	0.857	0.104	0.753
Specificity	0.917	0.017	0.900
Accuracy	0.903	0.035	0.868

Based on 31 patients with seven hemorrhagic events. Optimism was estimated by bootstrap resampling with 2,000 resamples; within each resample the entire modelling procedure was repeated, including selection of the Youden-optimal threshold, so that the correction accounts for the optimism arising from deriving and evaluating the cutoff in the same data. Leave-one-out cross-validation yielded an area under the curve of 0.911. Across resamples the Youden-optimal cutoff had a median of 9.8 mm^2 (interquartile range 9.8–16.4; 2.5th–97.5th centile 5.3–19.7) and 9.8 mm^2 was selected in 42.6% of resamples. Abbreviation: ROC, receiver operating characteristic.

Table S1(b). Comparison of prediction models for subsequent variceal hemorrhage.

Model	Penalized log-likelihood	AIC	c-statistic	LOOCV AUC
Initial RC signs alone	−9.375	22.75	0.866	0.750
Variceal CSA alone	−3.980	11.96	0.946	0.911
Initial RC signs + variceal CSA	−4.099	14.20	0.952	0.851

All models were fitted by Firth's penalized-likelihood logistic regression in the same 31 patients with seven hemorrhagic events. Adding CSA to the model containing RC signs improved fit (penalized likelihood ratio test $\chi^2 = 5.77$, df = 1, P = 0.016), whereas adding RC signs to the model containing CSA did not ($\chi^2 = 0.71$, df = 1, P = 0.401). The lowest AIC and the highest leave-one-out cross-validated AUC were both obtained with CSA alone, consistent with overfitting of the two-predictor model at 3.5 events per variable. Abbreviations: AIC, Akaike information criterion; AUC, area under the receiver operating characteristic curve; CSA, cross-sectional area; LOOCV, leave-one-out cross-validation; RC, red color.

Table S2. Tipping-point analysis of hypothetical hemorrhage outcomes imputed for the nine patients who did not achieve variceal eradication.

Assumed outcome scenario	OR per 1 mm^2 (95% CI)	AUC	P value
Most favourable (CSA ≥ 9.8 mm^2 bled; below did not)	1.550 (1.245–2.439)	0.961	<0.001
None of the nine bled	1.160 (1.047–1.322)	0.861	0.004
All nine bled	1.453 (1.198–2.046)	0.924	<0.001
Random assignment at observed event rate (22.6%)	1.170 (1.056–1.330)	0.861	0.002
Maximally adverse (CSA ≥ 9.8 mm^2 did not bleed; below bled)	1.110 (1.016–1.231)	0.787	0.020

Hemorrhage outcomes were not available for the nine patients who did not achieve eradication, but variceal cross-sectional area was measured in all nine (6.1, 7.1, 7.7, 8.4, 15.2, 16.0, 16.8, 21.6, and 29.6 mm^2). Outcomes were therefore imputed across the full range of possible scenarios and the univariate analysis was repeated in the combined cohort of 40 patients. Estimates were obtained by Firth's penalized-likelihood logistic regression, with confidence intervals and P values based on the profile penalized likelihood. For the random-assignment scenario, values are the medians of 500 replicate imputations. The imputed outcomes are hypothetical; this analysis is presented solely to characterize the robustness of the observed association to the missing data and no combination of imputed outcomes abolished it. Abbreviations: AUC, area under the receiver operating characteristic curve; CI, confidence interval; CSA, cross-sectional area; OR, odds ratio.

Table S3. Measurement-error sensitivity analysis of the association between variceal cross-sectional area and subsequent variceal hemorrhage under assumed levels of interobserver agreement.

Assumed ICC	Error SD (mm^2)	OR per 1 mm^2 (median)	AUC, median (2.5th–97.5th centile)	Replicates with $P < 0.05$
0.95	1.53	1.394	0.946 (0.893–0.976)	100%
0.90	2.23	1.377	0.940 (0.863–0.988)	100%
0.85	2.81	1.359	0.929 (0.842–0.988)	100%
0.80	3.34	1.338	0.923 (0.827–0.988)	100%
0.70	4.37	1.294	0.905 (0.798–0.988)	100%

Individual measurements from each examiner were not archived and a measured interobserver agreement coefficient could not be computed. A hypothetical second reading was therefore simulated by adding classical, non-differential measurement error to each recorded value, with the error standard deviation derived from the assumed intraclass correlation coefficient as $\sigma_e = \sigma \times \sqrt{(1 - ICC)/ICC}$, where σ is the observed standard deviation of cross-sectional area (6.68 mm^2). The entire analysis was repeated in 2,000 simulated replicates at each assumed level of agreement. This is a simulation and is not a substitute for measured interobserver agreement, which is identified in the Discussion as a requirement of the confirmatory study proposed. Abbreviations: AUC, area under the receiver operating characteristic curve; ICC, intraclass correlation coefficient; OR, odds ratio; SD, standard deviation.
